# The Role of Mesenchymal Stromal Cells and Their Products in the Treatment of Injured Spinal Cords

**DOI:** 10.3390/cimb45060329

**Published:** 2023-06-16

**Authors:** Lucia Slovinska, Denisa Harvanova

**Affiliations:** 1 Associated Tissue Bank, P.J. Šafárik University and L. Pasteur University Hospital, 040 01 Košice, Slovakia; denisa.harvanova@upjs.sk; 2 Department of Regenerative Medicine and Cell Therapy, Institute of Neurobiology Biomedical Research Center, Slovak Academy of Sciences, 040 01 Košice, Slovakia

**Keywords:** mesenchymal stromal cells, spinal cord injury, treatment, extracellular vesicles, secretome

## Abstract

Spinal cord injury (SCI) is a destructive condition that results in lasting neurological damage resulting in disruption of the connection between the central nervous system and the rest of the body. Currently, there are several approaches in the treatment of a damaged spinal cord; however, none of the methods allow the patient to return to the original full-featured state of life before the injury. Cell transplantation therapies show great potential in the treatment of damaged spinal cords. The most examined type of cells used in SCI research are mesenchymal stromal cells (MSCs). These cells are at the center of interest of scientists because of their unique properties. MSCs regenerate the injured tissue in two ways: (i) they are able to differentiate into some types of cells and so can replace the cells of injured tissue and (ii) they regenerate tissue through their powerful known paracrine effect. This review presents information about SCI and the treatments usually used, aiming at cell therapy using MSCs and their products, among which active biomolecules and extracellular vesicles predominate.

## 1. Introduction

At present, spinal cord injury (SCI) is a serious traumatic neurological situation leading to irreversible neurodegenerative changes with a major effect on the patient’s quality of life as well as on society. After SCI, mammals are unable to regenerate nerve tissue due to poor neuronal plasticity. SCI in humans is mainly caused by traumatic events, such as falls, car crashes, criminal acts, and sports injuries. The course of SCI depends on several factors, such as the site of spinal cord damage, the severity of the injury, and the age and condition of the patient. Nowadays, there is no effective method of treatment with the possibility of returning the patient to the original state of life before the injury. Accidental primary mechanical injury triggers a whole series of structural damages representing the primary phase of injury resulting in disruption of axons, blood vessels, and neural-cell membranes; massive necrotic cell death; and the breakdown of the blood–brain barrier [1]. As a result of damage to blood vessels, there is insufficient blood supply to the injured area. Immediately after that, the processes of the secondary phase of the damage begin, where nearby glial cells and nerve cells become injured due to the release of toxic chemicals from disrupted cells [2]. The secondary injury is a series of sequential changes that begins within hours of the SCI and may persist for more than 6 months after the initial damage. This cascade of uncontrolled pathophysiological processes results in swelling of the spinal cord, edema, hemorrhage, ischemia, the loss of ionic homeostasis, excitotoxicity, cell apoptosis, neuronal death, demyelination process, and cavity and glial scar formation with reactive astrogliosis [1,3], so the lesion becomes an increasingly hostile microenvironment after injury. Excitotoxicity is a highly disturbing process where neurotransmitters (glutamate and aspartate) play key roles. In the healthy spinal cord, the neurotransmitters are produced in negligible amounts, and they are part of the transmission of excitations. Under pathophysiological conditions, glutamate is over-expressed and effluxes from damaged spinal neurons, axons, and astrocytes, thereby overstimulating neighboring neurons. The overexcited cells release large amounts of calcium ions causing sequences of destructive events, including the production of free radicals. These highly reactive molecules cause free-radical-mediated lipid peroxidation leading to damage of cell membranes and other cellular components causing the apoptosis of remaining healthy neurons and glial cells in the surrounding areas. The first active players responding to damage are microglia cells, the main immune cells of the central nervous system. Microglia with low activation thresholds affect the morphology or function of other cells. Microglia cells after activation give rise to neurotoxic astrocytes and increase nerve tissue injury and neuronal loss [4]. Under physiological conditions, astrocytes provide essential physiological separateness and assistance for neurons. After SCI, astrocytes are activated, and some of them multiply rapidly via multiple inflammatory factors, including TNF, IFN-γ, IL-6, and IL-1β [5], and change to a reactive form. The role of reactive astrocytes in the early inflammatory response is to draft monocytes from peripheral blood to the injured site by producing several cytokines and chemokines (e.g., CCL2, CCL5, and CXCL8) [6]. Reactive astrocytes together with NG2+ oligodendrocyte progenitor cells and microglia participate in the formation of the glial scar, mainly via the production of extracellular matrix molecules, especially the chondroitin sulfate proteoglycans (CSPGs) [7]. The glial scar plays a dual role in the pathological process after SCI, both protective and inhibitory, where it separates damaged tissue from healthy tissue and at the same time prevents neuroregeneration in the form of overgrowth of new neurites and limits the therapeutic effects of drugs [3,8]. Therefore, one of the strategies for the treatment of SCI is aimed at scar formation. For example, the depletion of microglia cells leads to a decrease in the number of proliferating astrocytes and to the deterioration of the formation of astrocytic scars in the lesion area and prevents the neuroinflammatory response partly by inhibiting STAT3 phosphorylation in astrocytes [8]. The long-term effects of an acute spinal cord injury result from the location and severity of the injury, and the result of all these destructive events is that most patients remain paralyzed with severe health problems and great social burden. Some therapies focus on reducing the resulting inflammation response by administering drugs such as methylprednisolone with anti-inflammatory effects. Methylprednisolone is a drug with a demonstrable effect on improving motor and sensory functions in patients after SCI in the NASCIS-3 human trial [9]. Nowadays, there are several approaches in the treatment of a damaged spinal cord, with a goal to try to reduce the side effects of the damage and protect injured nerve tissue, but the golden standard treatment for patients with SCI is surgery and/or decompression, high doses of methylprednisolone, and rehabilitation.

Despite the fact that there is no effective treatment for the given condition, new procedures and ways of mitigating the impact of spinal cord damage on the patient’s overall condition are still being investigated. Cell therapies show great potential in the treatment of damaged spinal cords with a focus on controlling cell apoptosis, controlling the inflammation answer, and promoting nerve cell regeneration. Different types of cells have been used, but mesenchymal stromal cells (MSCs) and mononuclear progenitor cells (MNCs) were prevalent in registered clinical trials. In addition to these, fewer recent studies have been initiated with other cell types, such as Schwann cells (SC), neural stem cells (NSCs), neural progenitor cells (NPCs), olfactory ensheathing cells (OEC), and oligodendrocytes precursor cells (OPCs) [10]. Although NPCs show therapeutic worth, their translational potential is restricted by limited availability, immunologic problems, and ethical issues [11]. Transplantation of iPSC-based cells is also unsafe due to the potential for tumorigenicity, immunogenicity, and genetic and epigenetic cell abnormalities [12]. As for cell sources, the use of autologous cells exceeds allogeneic cells by three times [13].

## 2. Mesenchymal Stromal Cells (MSCs)

The term MSCs is used in connection with a mass population with remarkable secretion, immunomodulating, and homing features, and it is not interchangeable with another term, namely mesenchymal stem cells. The definition of mesenchymal stem cells is based on a population of stem cells in which the function of self-renewal and differentiation of progenitor cells is verifiable [14]. In compliance with the International Society for Cell and Gene Therapy (ISCT) and its definition, MSCs must meet the following conditions: (i) cells have the ability to adhere to plastic culture vessels, (ii) to express specific surface antigens (positive for CD73, CD90, or CD105, negative for CD45, CD34, CD14 or CD11b; CD79 alpha or CD19; and HLA-DR), (iii) ability to differentiate into mesenchymal trilineage in vitro [15]. MSCs are easily harvested and propagated with fewer ethical problems, low immunogenicity [16,17,18], and limited risk of developing tumors [19,20,21]. MSCs isolated from distinct sources show distinct characteristics, known as tissue-sources-associated heterogeneity. The most popular types of MSCs for the treatment of SCI are bone marrow mesenchymal stromal cells (BM-MSCs), adipose-tissue-derived mesenchymal stromal cells (AT-MSCs), and human umbilical cord mesenchymal stromal cells (hUC-MSCs). Other sources of MSCs are amniotic fluid and placentas, dental pulp [22,23], chorion, and Wharton jelly (WJ) [23,24].

MSCs are mainly isolated from human bone marrow from the upper iliac crest of the pelvis [25], which represents an invasive method of isolation for the patient. BM-MSCs, were among the first cells used to treat SCI [26]. The great advantage of AT-MSCs in comparison to BM-MSCs is that they can be isolated in a minimally invasive manner in large amounts without causing widespread damage. They also produce a cocktail of growth factors, extracellular matrix molecules, and proteases, thereby contributing to the promotion of angiogenesis and wound healing [27]. UC-MSCs in comparison to BM-MSCs and AT-MSCs exhibit higher proliferation, viability of differentiation ability, retardation of senescence, and higher anti-inflammatory effect. These results manifest that primitive UC-MSCs exhibit biological advantages in comparison to adult sources, making UCB-MSCs an appropriate model for clinical applications of cell therapy [28]. Transplanted hUC-MSCs after traumatic spinal cord injury facilitate functional recovery and are involved in reducing inflammation, scar formation, and astrogliosis [29]. Furthermore, transplantation of hUC-MSCs is shown to be dose-dependent, and better improvement is achieved with repeated placements [30].

MSCs have the capacity to rapidly proliferate, where in a few weeks they can multiply their number several thousand times. Though, as the number of in vitro passages increases, the quality of the cells themselves decreases. Large-scale in vitro cultivation causes cell senescence, which is associated with growth arrest and apoptosis. At the same time, MSCs from old donors have shown a reduced maximum length of life in comparison with cells from younger donors [31]. Depending on several culture parameters, including tissue source, isolation method, and media composition, the properties of human MSCs (hMSCs) can vary greatly [17].

## 3. MSC Therapy

Thanks to its regenerative and immunosuppressive properties, MSCs derived from adult tissues have become the cell of choice in the sphere of regenerative medical science. Currently, both autologous and allogeneic MSCs are in clinical use, where both have their advantages and disadvantages. When using allogeneic MSCs, donors can be selected according to the recipient, and the cells are ready for use, but there is a potential risk of an immune reaction with a specific immunological memory. MSCs have been supposed to have low immunogenicity. However, in vivo investigations are showing that allo-MSCs are not fully immune-privileged and likely cause an immune response resulting in rejection, they do elicit a humoral and cellular immune response in vivo, and allo-MSCs also appear to stimulate innate immune responses. However, there is no final clinical benefit of autologous MSCs versus allogeneic MSCs [32,33], and the complex view of MSCs’ allo-rejection is still evolving, and unanswered questions persist. Because allo-MSCs can persist for a short time after application, they may perform a protective and/or immunosuppressive mission in the short term, but they are less efficient in the long term [34]. For example, haplo-hematopoietic stem cell transplant with co-infusion of UC-MSCs justifies the effectiveness of UC-MSCs to prevent acute graft-versus-host disease in the treatment of children with high-risk acute leukemia [35].

Whereas autologous MSCs are easy to obtain, cells need some culture time to proliferate before they can be applied and they lack immune response [36], but auto-MSC therapy’s source of MSCs is limited.

**Figure 1:**
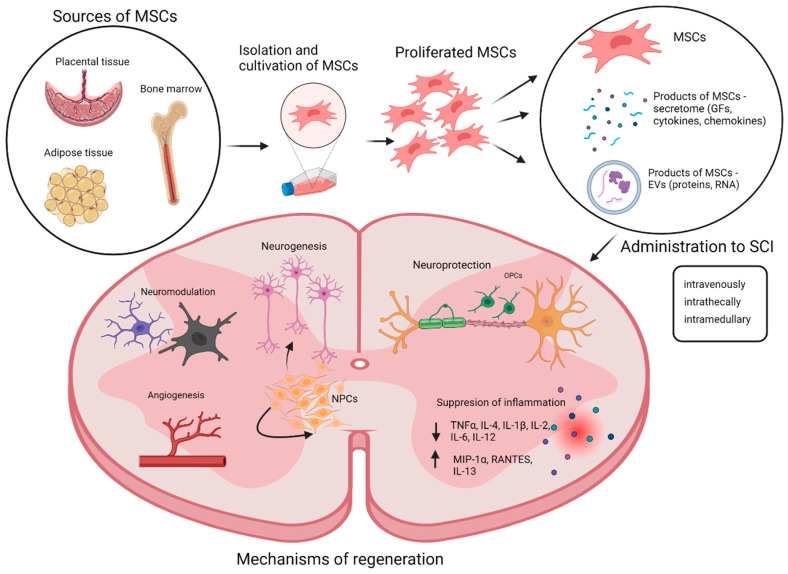
A schematic overview describing the isolation, application, and mechanisms of regeneration of mesenchymal stromal cells and their products in the injured spinal cord. EVs—extracellular vesicles, GFs—growth factors, MCSs—mesenchymal stromal cells, NPCs—neural progenitor cells, OPCs—oligodendrocyte progenitor cells. Created with BioRender.com.

The regenerative abilities of MSCs are different; it depends on whether the MSCs themselves are involved in the regeneration of injured tissue or the products they produce and send to their surroundings (Figure 1). MSCs are known to have the ability to replace the cells of the nervous system that have died due to the damage, reduce astrocyte proliferation, and rebuild the damaged nerve tissue via differentiation into neuron-like cells and glial cells and stimulation of neural stem cell proliferation [37,38].

BM-MSCs cultured under specific conditions, in the presence of EGF or BDNF, are capable of differentiating into cells that express several neural proteins and are similar to immature neurons or glial cells [39,40]. Lee et al. proved that human BM-MSCs differentiate into neuron-like cells by co-treatment with a highly specific ROCK inhibitor and CoCl_2_ [41] (Lee, 2010). Adult MSCs can express neuronal markers when they are co-cultured with cerebellar granule neurons. Eventually, MSCs can differentiate in vitro into excitable neuron-like cells with the ability to respond to different neurotransmitters such as GABA, glycine, and glutamate [42].

MSCs after gene modification, such as neural stem cells and spinal cord precursor cells, can be used to replace cells after SCI. For example, BM-MSCs can be induced into neural cells via the human brain-derived neurotrophic factor gene in a functionalized self-assembling peptide hydrogel [43]. AT-MSCs showed their neurotrophic features via the ability to express structural proteins that encode genes, mimicking the function of astrocytes, thus supporting the metabolism and functions of neurons in the central nervous system and the ability to differentiate into astrocytes [44].

After application, MSCs can also have an analgesic effect; they relieve neuropathic pain and support functional recovery [45]. The analgesic effect is caused by a reduction in the secretion of inflammatory factors IL-6 and TNF-α and up-regulation of GDNF production. In addition, the results from the work where they compared the effects of BM-MSC and UC-MSC transplanted after SCI confirm that the effectiveness of both sources was similar for alleviating the symptoms of neuropathic pain and for subsequent motor recovery after SCI [46]. However, the mechanism by which MSCs alleviate neuropathic pain caused by SCI after MSC transplantation is not fully elucidated. The mechanism may be associated with the activation of glial cells, reversible regulatory mechanisms between inflammatory/immune cells and microglia, and regulation of inflammatory factors after SCI. The effect of cell therapy in regeneration medicine depends on several factors, e.g., method of application, doses of administrated cells, and time frame of cell transplantation after injury.

### 3.1. MSC Doses, Method, and Time Frame of the Application

Upadhyayula et al. [47] reported, that in 2021, 18 clinical studies of stem-cell-based treatment for SCI were recorded as completed on clinicaltrials.gov (accessed on 18 May 2023), with up to 37 more participating patients, where the majority of data points to the fact that injection of stem cells into the spinal cord is considered harmless with minimal side effects. MSCs can be delivered to patients with SCI in several ways: intravenous, intrathecal, and direct intramedullary. Intravenous and intrathecal/lumbar punctures are routine procedures and predominate over intramedullary injection, which represents a new and challenging method [48]. Intravenous administration of cells has certain disadvantages, because intravenously administered MSCs are short-term [49], fragment after systemic administration in mice, acquire markers of apoptotic and phagocytic cells, and gather in the lungs and liver [50,51]. The intramedullary route allows the greatest concentration of stem cells to be provided to SCI patients. Saini et al. [52] found that intramedullary application of BM-MSCs administered intraoperatively during spinal decompression and fusion is safe and appropriate for patients with acute complete SCI. When comparing intraperitoneal and intravenous administration of BM-MSCs, the results showed that the two approaches have similar therapeutic effects on SCI [53].

Intranasal administration also represents one of the possibilities of cell application. BM-MSCs after intranasal administration can migrate to the injured spinal cord and support the reduction of lesions and the recovery of hindlimb motor function. However, when compared with intrathecal administration, intranasal application does not have such a significant healing effect [54].

Although various methods and techniques of cell administration are being tested, such as intravenous, transarterial, nasal, intraperitoneal, intrathecal, and intramedullary injections, the optimal method of administration has not yet been determined. However, all these methods are shown to be relatively safe and without major problems [55].

In terms of many applications, some SCI studies show improvement after a single injection of stem cells, but investigations with multiple injections spread over time have greater improvement in outcomes. Another important factor affecting the course and effect of SCI treatment with transplanted cells is the number of cells because if an insufficient number of cells are implanted, the treatment itself will be ineffective. In different studies, the number of applicated cells was in the range from 1 × 10^5^ to 40 × 10^7^, and the time frame was 8 weeks post-injury. There was no consistent relationship between cell count and outcome in the mentioned studies [47]. Additionally, an early BM-MSC transplantation, within one week after SCI, contributes to the reduction of the acute inflammatory response after SCI [55]. Furthermore, cell transplantation from 1 week to 2 weeks after SCI is also successful in restoring nerve function because a large amount of neurotoxins are produced at this time in the damaged area, which can reduce the chance of survival of the transplanted cells [56]. Application of MSCs after a longer period after SCI even contributed to the neuroprotective effects by controlling the stages of microglia and BDNF in the spinal cord [57]. It is proven that intrathecal implantation of 1 × 10^6^ hUC-MSCs/kg is an alternative approach for the treatment of subacute SCI with desirable therapeutic results in rats, whereas GABA_A_ receptors are potential candidates for a therapeutic focus [58].

Vaquero et al. [59], in a phase 2 clinical study in chronic SCI patients with three intrathecal transplantations of 100 × 10^6^ MSCs, concluded that therapy was well accepted, without any negative effects corresponding to MSC application. Patients reported changeable clinical progress in sensibility, motor strength, spasms, spasticity, neuropathic pain, sexual function, or sphincter dysfunction, irrespective of the level or grade of injury, age, or time since the SCI.

In addition, intrathecal transplantation of Wharton-jelly-MSCs (WJ-MSCs) isolated from the human umbilical cord (hUC) is a safe procedure. A single infusion of WJ-MSCs intrathecally applied to patients with chronic complete SCI resulted in sensory improvement in the segments located near the site of injury [24]. Even multiple administrations of MSCs did not cause adverse events. Even in a patient with chronic complete spinal cord injury who received six doses of MCSs, there was an improvement in sensory and motor functions, which resulted in a better quality of life for the patient himself [60].

Similarly, Yang et al. [61] present a protocol demonstrating that intrathecal/subarachnoid application of allogeneic hUC-MSCs at a dose of 10^6^ cells/kg once a month for four months in patients suffering from SCI is harmless and effective and resulted in a marked improvement in neurological dysfunction and restoration of quality of life. After intravenous administration, MSCs affect not only damaged nerve tissue, but also non-directly damaged tissue. Applied MSCs activated compensatory mechanisms that contributed to circuit reorganization and alternate routes, and with an increased axonal network in corticospinal tracts, they could contribute to functional recovery beneath the injury [62].

In addition, our study, where we characterized the presence and the expression profile of several proteins and some neuro markers in the serum and cerebrospinal fluid of a SCI patient cured with one dose of autologous BM-MSCs applicated intrathecally, led to an eminent decrease in the levels of neuro markers, cytokines, chemokines, and growth factors that participated in the inflammatory response [63].

Important factors are the time elapsed from injury to treatment, the duration and chronicity of treatment, and the actual delivery of cells. Depending on the dose of cells administered and the time of administration after SCI, studies focusing on acute or subacute SCI cases report a more dramatic recovery of function in comparison to chronic SCI [47,64,65].

### 3.2. MSCs with Biomaterials and Scaffold

Because MSCs have a low ability to survive and engraft after transplantation in the lesion site after SCI, different support materials have been used for better cell fixation and to promote their therapeutic efficacy at the site of damage. The focus has mainly been on biodegradable natural materials including gelatin, hydrogels, alginate, agarose, collagen, and chitosan. The scaffold must not be toxic and cause injury such as inflammation and should protect the transplanted cells, to minimize their distribution and to promote their survival and proliferation when implanted into the injured site. Transplanted cells mixed with biomaterials can be injected into the lesion site, which can produce trophic factors necessary for neuroregeneration and promote axon repair.

These biomaterials either provide mechanical support for transplanted MSCs or are also enriched with various growth factors, which are gradually released into the environment and help the regeneration process in the damaged spinal cord [66,67].

Alginate, a hydrophilic polysaccharide, is primarily derived from brown seaweed and bacteria. Alginate-based hydrogels were used to investigate the promotion of controlled axonal regeneration after implantation into the acute cervical spinal cord lesions in adult rats. No inflammatory response was induced, while the hydrogel promoted axonal regeneration across the scaffold [68]. Blaško et al. [69] showed that the application of alginate hydrogel enriched with MSCs creates a supportive environment for endogenous regeneration processes, which are manifested by increased axonal outgrowth reflected in a significant increase in the number of axons positive for the GAP-43 marker.

Chitosan is a polysaccharide produced by the deacetylation of chitin, which is contained, for example, in the exoskeletons of crustaceans. A chitosan scaffold promotes cell adhesion. Its porous structure permits cell propagation and supply of nutrients, and it is effective for angiogenesis, which is important for the regeneration of soft tissues [70]. It can be easily processed and manufactured in different forms or shapes, which is an important feature for SCI treatment, as we can control the direction and orientation of neurons growth in this way [66]. Chitosan-based hydrogels do not affect MSCs’ viability, and encapsulated MSCs have the ability to secrete MSC vesicles as well as retain their anti-oxidant properties [71]. 

It is proven that animals implanted with nano-hydrogel (gold-nanoparticle-loaded Agarose/Poly (N-isopropyl acrylamide)) combined with BM-MSCs have faster recovery of motor function after post-operative surgery, in comparison to the other implanted animal groups [30]. The delivery of an alginate scaffold enriched with epidermal growth factor and basic fibroblast growth factor, which was regularly released into the central lesion site, significantly increased the sparing of spinal cord tissue and increased the number of surviving neurons with the acceleration of neovascularisation and significantly improved functional recovery in SCI groups receiving the alginate scaffold [72].

Furthermore, the transplantation of the gelatin sponge scaffold modified with NT-3 inhibits local inflammation, enhances nerve fiber regeneration, attracts the migration of cells in host tissue into the injury/graft position to form a myelin sheath and blood vessels, and improves neural conduction in the canine with spinal cord injury [73].

Collagen is the most popular protein, and it is used in the medical field, among others, as a scaffold. Different kinds of MSCs have been used in conjunction with collagen, but BM-MSCs have been the most studied. BM-MSCs were transplanted in combination with collagen into either an acute [74,75] or chronic [76] spinal cord injury model. Combined collagen and MSC transplantation had a beneficial effect on neuroprotection and neurite direction. Collagen offered a support matrix for cells and generated a friendly microenvironment for the regeneration of axons by inhibiting the formation of glial scars. At the same time, MSCs modulated M1 to M2 shift in the post-SCI microenvironment with higher expression of type M2 to form anti-inflammatory surroundings. The combined application of collagen and MSCs might be a noticeable strategy for SCI treatment in the future.

After MSC transplantation with a linearly ordered collagen scaffold, NeuroRegen scaffold, a marked improvement of sensory and motor function was observed in two patients with acute complete SCI. That was the first time that MSC-enriched NeuroRegen scaffolds were transplanted to treat patients with acute SCI who were judged as having ‘complete’ injury with a stringent method [64]. Graphene oxide (GO)-based materials are being pushed to the force in biomaterial and tissue engineering thanks to their exceptional chemical, mechanical, and electrical abilities and easily modifiable structure. A preliminary in vitro study on mesenchymal stromal cells showed that the created nanocomposites containing chitosan and polyethylene glycol are not only non-toxic but also raise the growth of cells [77].

## 4. Paracrine Effect of MSCs

At the beginning of using cells to regenerate damaged tissues, it was assumed that MSCs were capable of generating three principal kinds of cells of the nervous system (neurons, astrocytes, and oligodendrocytes) for the replacement of injured cells after transplantation. However, a growing number of studies figured out that this neurological improvement may not be due to MSCs’ ability to differentiate into neuronal cells, but rather, the paracrine effect of MSCs is more significant in the regeneration of the damaged spinal cord. MSCs actively secrete biomolecules that act either on themselves (autocrine function) or neighboring cells (paracrine function). Thanks to their homing properties, MSCs are able to migrate to the site of injured tissue, where they are attracted by various inflammatory or chemotactic factors [78], so MSCs and their secretome may affect the microenvironment of the spinal cord after injury, as well as endogenous cells already present at sites of injury. However, in vivo, studies found poor engraftment and survival of MSCs when injected into SCI due to the formation of the glial scar, with chondroitin sulfated proteoglycans (CSPGs) as an integral element. Wood et al. [79] demonstrated that MSCs are resistant to CSPG exposure, but that CSPGs have a significant effect on their regenerative activity due to inhibited MSC adhesion. CSPGs reduced their pro-adhesive and proliferative effects on endothelial cells, but the lifespan of MSCs themselves was not affected. A possible explanation is that transient changes observed in MSC morphology occurred, changing from a fibroblastic shape to a spherical cell shape. In addition, CSPGs inhibited the differentiation of BM-MSCs as well as WJ-MSCs into neuron-like cells via the Rho/ROCK pathway. Therefore, the use of ROCK inhibitors may be effective for neuronal regeneration during cell therapy by hindering the inhibitory effect of CSPGs on transplanted MSCs in injured tissues [80]. The discovery enlarges the understanding of how the post-SCI wound microenvironment may attenuate the beneficial effects of MSC transplantation.

There is increasing evidence that MSCs produce various neurotrophic factors as well as chemokines and cytokines in vitro and in vivo involved in neuroprotection, immunomodulation/suppression of inflammation, apoptosis, neurogenesis, and angiogenesis (Table 1). MSCs can regulate adaptive immune cells, such as altering B cell proliferation and differentiation, thereby inhibiting T cell proliferation, as well as being able to control the immune response from innate immune cells including monocytes, macrophages, their polarization, phagocytosis, and metabolism [81,82]. Human cardiac AT-MSCs can reprogram macrophages into a reparative, anti-inflammatory M2-like phenotype. At the same time, AT-MSCs increase the secretion of anti-inflammatory and angiogenic cytokines (IL-10 and VEGF) and reduce the secretion of inflammatory cytokines (IL-1α, TNFα, IL-17, and INF-α). Reprogramming macrophages from M1 to M2 phenotype via MSCs could be useful to manage and figure out the inflammation reaction after tissue injury [83]. MSCs can polarize monocytes or M1 macrophages into M2-type macrophages through direct cell-to-cell contact or by secreting soluble factors such as PGE2, TGF-β, TSG-6, CCL2, and CXCL12 via the modification of diverse metabolic pathways, for example, glycolysis, oxidative phosphorylation, Krebs cycle, and fatty acid oxidation [84,85].

Furthermore, other studies found that acute transplantation of MSCs into SCI rat contusion models modified the inflammatory environment by up-regulating the number of alternatively activated M2 macrophages and down-regulating the number of classically activated M1 macrophages. This shifting of the macrophage phenotype occurs with increased levels of IL-4 and IL-13, and decreased levels of TNF-α and IL-6, which might support the recovery of motor function, increase the permissive environment for axonal extension and myelin sheath, as well as promote less glial scar formation following injury [86]. This anti-scarring property could also help prevent secondary injuries in the spinal cord after the initial trauma.

The results of animal studies show that intrathecal application of MSCs lowers the inflammatory reaction and apoptosis via reduced levels of TNFα, IL-4, IL-1β, IL-2, IL-6, and IL-12 and accelerates the levels of MIP-1α and RANTES [87]. Human MSCs produce factors eminent for mediating the overgrowth of axons and regeneration after SCI, but batches of MSCs from different donors vary widely [88]. It is not yet clear which factors or combinations of factors are essential in promoting recovery. For significantly effective therapeutic success, it is necessary to better and more precisely define and characterize the MSCs’ secretome.

### 4.1. Secretome

MSCs’ secretome can be interpreted as a complete mixture of MSC-derived bioactive molecules (soluble proteins, cytokines, chemokines, nucleic acids, lipids, and extracellular vesicles), an important signaling mechanism to affect other cells, that can endorse tissue repair and regulate inflammatory and immune response [89,90]. In recent years, there has been a growing interest in using MSCs’ secretome as a cell-free therapy for SCI. This method avoids many of the problems caused by stem cell transplantation, such as ethical issues, immune rejection, and the potential for tumor formation. In SCI, the MSCs’ secretome has been shown to support the survival of damaged neurons, increase the production of new nerve cells, and promote the growth of new blood vessels. Administration of MSCs’ secretomes represents a new, cell-free therapeutic perspective for the reduction of inflammatory and degenerative diseases. By using their secretome, MSCs can encourage the propagation and differentiation of different cell types, including themselves. 

MSCs have the ability to secret various soluble biomolecules with anti-inflammatory effects, including TNF-β1, IL-13, IL-18, binding protein, ciliary neurotrophic factor (CNTF), neurotrophin-3 (NT-3), IL-10, and IL-27. At the same time, MSCs can affect the cytokine generation of the organism where they were transplanted and thereby encourage or inhibit the production of anti-inflammatory factors (such as IFN-γ and IL-10) [91]. The microenvironment also appears to influence the effects of the MSC secretome. Exposure of MSCs to CSPGs reduced the extent to which MSC secretome supported some aspects of angiogenesis [79].

Kiselevskii 2021 et al. [92] studied the profile of the cytokines of MSC culture derived from bone marrow and demonstrated the intense release of IL-6, IL-8, and chemokine MCP-1, which are involved in the pathogenesis of cytokine storm and graft-versus-host disease. MSCs can also reduce inflammation by instructing microglia to a beneficial phenotype mainly through the CX3CL1/CX3CR1 signaling pathway [93].

BM-MSCs are able to inhibit TLR4-mediated signaling and reduce interleukin-1β (IL-1β) and tumor necrosis factor-α (TNF-α) expression to attenuate the inflammatory reaction caused by SCI and optimize neurological function in rats [94].

Delfi et al. [95] investigated the paracrine effects of human and canine MSCs cultivated under the same conditions and found that MSCs from both species have similarly expressive trophic effects on neuronal and endothelial cells. Conditioned medium derived from human embryonic stem-cell-derived MSCs is effective in the promotion of neurogenesis, either by mediating the release of angiogenesis and neurotrophic factors or by inhibiting the inflammatory reaction and production of apoptotic factors [96]. Szekiova et al. [97] analyzed the proteomic profiles of BM-MSC-conditioned media obtained by conditioning for different times and their effect on astrocyte migratory response and oligodendrocyte density. They found a progressive increase in the concentration of neurotrophic factors (NGF and VEGF) and a more powerful effect on regenerative processes in neural-cell cultures. In addition, secretome from adipose-tissue-derived MSCs plays an important role in neurological applications, especially in remyelination processes [98]. MSCs produce limited amounts of neuro-regulatory proteins such as BDNF and β-NGF, which elevate survival and induce neurite formation in primary nerves from the lumbar spine [99].

MSCs and MSC-derived bioactive molecules may depend on more systemic elements than those occurring within the CNS itself. One must be careful and correctly interpret the results obtained with different model systems to inspect the effects of transplanted cells and secretion on the CNS. Understandably, there is a different effect regarding how MSCs and the secretome they produce may influence the microenvironment of the spinal cord following the injury within an in vitro cell culture or a slice-culturing technique cultivated in the absence of corresponding circulatory models. These static conditions could change the answer of resident cells to the MSCs, because of no existing influx of immune cells or delivery of nutrition, as well as no system that would liquidate waste [100]. Although the MSC secretome also holds significant promise as a potential therapeutic strategy for the therapy of SCI, other studies are needed to fully understand the molecular mechanisms underlying its therapeutic benefits and to optimize its clinical administration.

**Table 1:** Mesenchymal stromal cell major neuroprotective and angiogenic factors.

Factor	Effect	References
vascular endothelial growth factor (VEGF)	supports angiogenesis, enhances the autophagic flux and reduces inflammation reaction, promotes neuronal survival	[101,102]
basic fibroblast growth factor (bFGF)	modulates neural progenitor proliferation and differentiation	[103]
nerve growth factor (NGF)	promotes angiogenic activity and reduces apoptosis, promotes neuronal cell survival and neuritogenesis	[99,104,105]
brain-derived neurotrophic factor (BDNF)	neuroprotective potential on primary auditory neurons increases neuroregeneration and synapses using BDNF-dependent mechanisms ppAKT and ppERK 1/2	[99,106,107]
hepatocyte growth factor (HGF)	mediates functional recovery and remyelination through HGF/cMet signaling pathway	[108,109]
chemokine ligand 1 (CX3CL1)	activation of microglia to a neuroprotective phenotype	[93]

### 4.2. Extracellular Vesicles (EVs)

The study of EVs is an active and rapidly developing field of research. The International Society for Extracellular Vesicles (ISEV) according to Minimal Information for Studies of Extracellular Vesicles 2018 (MISEV2018) ratifies ‘extracellular vesicle’ (EV) as a general term for particles naturally released from the cell that is bounded by a lipid bilayer without the possibility of replication, i.e., they do not have a functional nucleus [110]. We recognize different subtypes of EVs based on diameter, function, and source. Exosomes are membranous EVs with a size range of ~40 to 160 nm (average ~100 nm) in diameter with an endosomal origin and are formed by the internal budding of the multivesicular body membrane. Exosomes, a subset of EVs, can be continuously secreted into the extracellular space by most cell types; are found in different body fluids such as blood, urine, serum, amniotic fluid, umbilical cord, cerebrospinal fluid; and can be separated using different methods, e.g., centrifugation, size-exclusion chromatography, and precipitation. The properties of released EVs are highly determined by the origin, type, and condition of the cells from which they are derived. They carry vital information, major functional effector substrates, and macromolecules from their source of origin, so they are important mediators of cellular communication involved in many normal and pathological processes [111,112,113,114]. EVs regulate intercellular communications by transporting nucleic acids, lipids, proteins, and other signaling molecules. EVs can influence the response of recipient cells via several mechanisms, namely, EVs can directly modulate the phenotype and the functions of target cells through receptor–ligand interactions or fuse with target cells and modify their activity by delivering mRNAs, lipids, and miRNAs after internalization [115,116,117]. The most attractive point of EVs is the replacement of stem cells with a new generation of biological treatment methods. MSC-EVs can be isolated from different types of MSCs: human umbilical cord MSCs [111], bone marrow MSCs [118], and human placenta MSCs [119]. MSC-derived EVs can be easily obtained from the culture medium of MSCs with resulting concentrated and purified EV products. This fact makes them an attractive and cost-effective alternative to MSC-based therapy [120]. Effective delivery of a functional drug to the damaged spinal cord is considered one of the biggest missions because most substances have problems passing through the blood–spinal cord barrier (BSCB) and achieving the site of the injured spinal cord. EVs can also cross the blood–brain barrier but at varying rates and with various vesicular-mediated mechanisms [121]. It is for this reason that choosing the method of EV application is important in therapy. For example, a very effective application is an intranasal administration of BDNF-enriched EVs in the treatment of cerebral ischemia. The therapeutic effect is probably explained by activation of BDNF/TrkB signaling [122].

The connection of biomaterial scaffolds with EVs effectively transports and fastens EVs to the site of injury and optimizes their neurogenerative effects. Various forms of biomaterial scaffolds are used to offer a supportive microenvironment while EVs continuously produce growth factors and other nutrients, such as 3D-printed scaffolds, hydrogels, and alginate scaffolds [123]. New materials and innovative implantation strategies are constantly being developed to optimize the therapeutic effects of EVs. For example, the administration of exosomes derived from MSCs attached to a peptide-modified adhesive hydrogel exhibits effective retention and prolonged release in the host neural tissues and induces important nerve recovery and urinary tissue preservation by effectively attenuating inflammation and oxidation [124]. The effectiveness of EVs also increases if they are applied in a collagen gel, which was shown in the repair of peripheral nerves in the early stages (15–30 days) of acute injury of peripheral nerves [125].

MSC-derived EVs can provide therapeutic effects that are comparable to their parent cells because they have significant immunosuppressive properties and are able to go through the blood–spinal cord barrier (BSCB) [126]. As with their parent MSCs, EVs can regulate inflammation and induce and promote neuroprotection, neurogenesis, and angiogenesis. Sung et al. [127] intravenously transplanted EVs from human epidural adipose-tissue-derived MSCs in SCI-induced rats with the results that EVs had therapeutic potential and improved locomotor function by regulating the inflammatory response. EVs can control the immune reaction by impacting the gene expression and various signaling pathways in the recipient’s cells, especially through miRNA delivery. miRNAs are small, highly conserved non-coding RNAs that manage the gene expression in the posttranscriptional phase by targeting mRNAs. MSC-derived EVs containing miR-145-5p mitigate the inflammatory response in SCI by controlling the TLR4/NF-κB signaling pathway [128]. Exosomal miR-125a derived from BM-MSC has neuroprotective impacts by targeting and negatively regulating interferon regulatory factor 5 (IRF5) production in rats with SCI [118]. Deng et al. [129] found that miR-136-5p influences the inflammatory reaction after SCI by directing the IKKβ/NF-κB/A20 signal path. Xin et al. [130] provide the first evidence that MSCs can regulate neurite growth via the transmission of miR-133b into neural cells via exosomes by communicating with brain parenchymal cells.

MSC-derived EVs exhibit neuroprotective features by lowering the neuroinflammatory reaction induced by cerebral ischemia and supporting the angiogenic process [131]. EVs can act as potent delivery carriers and can be loaded with various cargo. Ran et al. [132] used autologous exosomes from plasma in vivo as delivery vehicles filled with neuron-targeting (RVG) and axon-growth-supporting peptides (ILP and ISP). In the end, when they penetrated the target sites of damage, they did not cause any post-treatment immunogenicity. The administration induced strong axonal regeneration and a new generation of intraspinal circuits below the level of the lesion in the SCI model of nude mice.

Angiogenesis is a key stage in the process of tissue healing and repair. The angiogenic process at sites of injury offers a distinct vascular delivery that can provide different cell types and bioactive components that trigger and promote tissue repair. MSCs closely communicate with endothelial cells, so MSCs act as facilitator cells concerning angiogenesis. EVs released from human adipose-derived MSCs (hadMSC-EV) are taken up by endothelial cells and prominently elevate the angiogenic process in vitro and in vivo. They contain a collection of angiogenic factors that make endothelial cell migration easier and elevate the activation of other angiogenic growth factors and signaling molecules, such as MFGE8, ANGPTL1, and thrombopoietin [112]. Liang et al. [133] recognized that MSC-Exo and exosomal transferred miR-125a elevate the angiogenic process through the activation of endothelial tip cell formation, suggesting a novel role of exosomal transferred miR-125a in controlling endothelial tip cell specification by altering its target delta-like ligand 4.

## 5. Clinical Studies

Clinical applications using MSCs and their products for SCI therapy have become an important field of research over the last years. Currently, 37 clinical trials involving MSCs in spinal cord injuries are listed on www.clinicaltrials.gov, accessed on 9 May 2023. In terms of cell type, autologous BM-MSCs are mostly administered, followed closely by UC-MSCs and AT-MSCs, with a predominance of chronic SCI damage in the thoracic and cervical area and intrathecal/subarachnoid/intramedullar administration. The safety of autologous transplanted BM-MSCs was measured via the absence of neuronal changes, infections, or increased intracranial tension and by monitoring for any abnormal growth or tumor formation. The results of a phase I/II controlled single-blind clinical trial study, where chronic cervical and thoracic SCI patients were treated with autologous BM-MSCs, showed that BM-MSC treatment in combination with physical therapy showed functional improvements with a higher rate in thoracic SCI patients. At 18 months post-treatment, 46% cases showed sustained functional improvement [134]. Furthermore, the results from a phase I clinical trial, where BM-MCSs were applicated via multiple routes, directly into the spinal cord, directly into the spinal canal, and intravenously, to patients with acute or chronic SCI showed morphological changes in the spinal cord of some patients and improvements in ASIA, Barthel (quality of life), Frankel, and Ashworth scoring, with no tumor formations and no cases of infection or increased pain [135]. In another study, which is still collecting and evaluating results, patients with complete or incomplete cervical, thoracic, and thoracolumbar SCI received four subarachnoid administrations of allogeneic hUC-MSCs with the delivery dose of 1 × 10^6^ cells/kg per subject with an interval of one month between each administration (NCT02481440). It seems that research is moving in this direction regarding the possible application of MSCs, i.e., administration in multiple doses and directly to the site of damage. It must not be forgotten that different clinical trials vary regarding the number of patients and with regard to the type and course of SCI. They also differ in the type of administered cells, the manner of application, and quantity. Therefore, it is difficult to compare them and to choose and determine one adequate and effective method of treatment. Therefore, further clinical trials are needed to investigate the clinical efficacy of the therapy both of the cells themselves and of their products.

## 6. Conclusions

In conclusion, mesenchymal stromal cells are a type of adult cells that have shown great potential in treating various neurological disorders, including spinal cord injury. However, not only are MSCs themselves in the sights of scientists, but scientists also are especially interested in their products, such as the mix of bioactive factors they release into their immediate surroundings as well as the extracellular vesicles that play an important role in communication between neighboring cells.

Further studies are needed to fully understand the molecular mechanisms underlying their therapeutic effects. This field of interest represents a challenge for further research with the potential for diagnostic and therapeutic applications and translations of these findings into clinical practice. It is clear that SCI is not a static disease and treatment at multiple levels is needed to achieve better outcomes. Although much work remains in this area, the future is shining.
